# Cloning and functional expression in *E. coli* of a polyphenol oxidase transcript from *Coreopsis grandiflora* involved in aurone formation^☆^

**DOI:** 10.1016/j.febslet.2014.07.034

**Published:** 2014-09-17

**Authors:** Cornelia Kaintz, Christian Molitor, Jana Thill, Ioannis Kampatsikas, Claudia Michael, Heidi Halbwirth, Annette Rompel

**Affiliations:** a Universität Wien, Fakultät für Chemie, Institut für Biophysikalische Chemie, Althanstraße 14, 1090 Wien, Austria; b University of Technology Vienna, Institute of Chemical Engineering, Getreidemarkt 9, 1060 Vienna, Austria; c University of Vienna, Department of Analytical Chemistry, Währinger Straße 38, 1090 Vienna, Austria

**Keywords:** AmAS1, aureusidin synthase from *Antirrhinum majus*, AUS, aurone synthase (protein), *cgAUS*, aurone synthase from *Coreopsis grandiflora* (gene), DTT, dithiothreitol, *EFα*, *elongation factor α*, IPTG, isopropyl-β-d-thiogalactopyranosid, LC, liquid chromatography, LC/MS, liquid chromatography/mass spectrometry, nanoUHPLC–ESI-MS/MS, ultra high performance liquid chromatography–electrospray tandem mass spectrometry, PHC, 2′,3,4,4′,6′-pentahydroxychalcone, PPO, polyphenol oxidase, *RuBisCO*, *ribulose*-*1*,*5*-*bisphosphate carboxylase/oxygenase*, SB, super broth media, THC, 2′,4,4′,6′-tetrahydroxychalcone, Type-3 copper, Polyphenol oxidase, Aurone synthase, Heterologous expression, 4-Deoxyaurone, *Coreopsis grandiflora*

## Abstract

Polyphenol oxidases are involved in aurone biosynthesis but the gene responsible for 4-deoxyaurone formation in *Asteraceae* was so far unknown. Three novel full-length cDNA sequences were isolated from *Coreopsis grandiflora* with sizes of 1.80 kb (*cgAUS1*) and 1.85 kb (*cgAUS2a*, *2b*), encoding for proteins of 68–69 kDa, respectively. *cgAUS1* is preferably expressed in young petals indicating a specific role in pigment formation. The 58.9 kDa AUS1 holoproenzyme, was recombinantly expressed in *E. coli* and purified to homogeneity. The enzyme shows only diphenolase activity, catalyzing the conversion of chalcones to aurones and was characterized by SDS–PAGE and shot-gun type nanoUHPLC–ESI-MS/MS.

## Highlights

• The first *PPO* gene (*cgAUS1*) involved in 4-deoxyaurone formation is identified.
• AUS1 is expressed as latent pro-enzyme in *E. coli* and purified to homogeneity.
• Diphenolase activity of AUS1 pro-enzyme is proven using SDS as an activation agent.
• Gene expression studies suggest a physiological role for AUS1 in aurone formation.

## 1 Introduction

Polyphenol oxidases (PPO) are an important class of type-3 copper enzymes. PPOs catalyze the oxidation of *o*-diphenols to highly reactive *o*-quinones that polymerize rapidly. Thus, PPOs are involved in the formation of pigments leading to browning of fruits and vegetables but can also catalyze distinct steps in the biosynthesis of secondary metabolites such as betalains and aurones [1], [2]. Enzyme nomenclature differentiates between tyrosinase (monophenol, *o*-diphenol: oxygen oxidoreductase, EC 1.14.18.1), catechol oxidase (CO, *o*-diphenol:oxygen oxidoreductase, EC 1.10.3.1), aureusidin synthase (2′,4,4′,6′-tetrahydroxychalcone 4′-*O*-β-d-glucoside:oxygen oxidoreductase, EC 1.21.3.6) and laccase (benzenediol:oxygen oxidoreductase, EC 1.10.3.2) [2]. Tyrosinases, a class of bifunctional PPOs, catalyze the conversion of phenols to *o*-diphenols under oxygen consumption, called monophenolase activity. The subsequent oxidation of *o*-diphenols to *o*-quinones, catalyzed by tyrosinases and catechol oxidases, is referred to as diphenolase activity.

PPOs frequently undergo post-translational processing. Typically plant PPOs contain a N-terminal chloroplast transit peptide [3], [4]. According to Tran et al. [3] and van Gelder et al. [4] the plant N-terminal transit peptides of PPOs are predicted to be chloroplast proteins. The core domain of about 39 kDa harbors a highly conserved copper binding site relevant for the catalytic activity and a C-terminal domain of about 18 kDa, cleaved during proteolytic processing [3], [5]. Effects of activators on the latency of the pro-enzyme (containing the core domain and the C-terminal domain) are summarized in a review by Yoruk and Marshall [6].

Aurones are yellow flower pigments. Two aurone types can be distinguished, 4-hydroxyaurones which are accumulated in *Antirrhinum majus* (old Scrophulariaceae, now Plantaginaceae) and *Helichrysum bracteatum* (Asteraceae), and 4-deoxyaurones which are found in many Asteraceae species, where they frequently co-occur with carotenoids [7]. Aurone formation is one of the rare examples of an involvement of PPOs in an anabolic pathway [1], [8], [9]. Formation of yellow 4-hydroxyaurone pigments in *A. majus* is catalyzed by aureusidin synthase, a bifunctional PPO homolog, which catalyzes two reactions, the hydroxylation and the oxidative cyclization of PHC and THC into aureusidin and bracteatin [8], [10]. Mature (active) aureusidin synthase was purified and characterized from *A. majus* petals [8]. Although a corresponding cDNA clone, expressed in the petals of aurone-containing varieties, was identified [8], studies on recombinantly expressed aureusidin synthase have not been reported so far.

Previous studies have shown that the formation of the more common 4-deoxyaurones differs from 4-hydroxyaurone biosynthesis in various aspects. Most notably, it was suggested that a catechol oxidase type enzyme rather than a tyrosinase type enzyme is involved [11] and that a specific biochemical background enables accumulation of aurones pigments. Molitor et al. (2014, submitted to FEBS) purified AUS from petals of *Coreopsis grandiflora* and demonstrated a hitherto unique unknown structural feature (disulfide linkage between C-terminal domain and main core) of the enzyme, that could be related to a more specialized physiological role compared to common PPOs. Although aurone synthase [11] is precisely an oxidase and not a synthase, the commonly used term aurone synthase better reflects the enzyme’s physiological relevance and its involvement in the anabolic pathway leading to the formation of aurone pigments. This work focuses on the (i) isolation of an *cgAUS* cDNA clone from a 4-deoxyaurone accumulating plant and (ii) investigation of functional activity of a recombinant PPO involved in aurone formation. The availability of the first *cgAUS* cDNA clone will allow studying the assumed differences between 4-hydroxy- and 4-deoxyaurone formation at the molecular level and is targeted towards revealing a biochemical pathway.

## 2 Materials and methods

### 2.1 Plant material & chemicals

*C. grandiflora* cv. Early sunrise was obtained from Volmary (Münster, Germany) and cultivated from April 2011 in the experimental field Augarten (1020 Vienna) of the University of Vienna. Flowers, leaves and stems from at least 10 plants were harvested, shock-frozen in liquid nitrogen and stored at −80 °C. Petal stages are shown in Table 1.

**Table 1:** Dependence of AUS activity and pigment accumulation on the stage of flower development; FW = fresh weight, s = seconds. *Note:* yellow pigmentation of *C. grandiflora* is caused by chalcones, aurones and carotenoids.

Tissue	Protein content [μg/10 μl]	Specific activity [nmol/(s * mg total protein)]	Yellow pigments [mg sulfuretin equivalents/g FW]	Ratio aurone:chalcone
Ray petal stage 1 closed buds 0.5 cm petal length	22	0.2	22.4	0.41
Ray petal stage 2 closed buds 0.9 cm petal length	22	0.1	23.7	0.27
Ray petal stage 3 opening flowers 1.2 cm petal length	18	0.5	15.9	0.23
Ray petal stage 4 open flowers 3.5 cm diameter	7	0.9	10.8	0.14
Disc petal stage 1 closed buds 0.5 cm petal length	17	0.1	7.7	0.70
Disc petal stage 2 closed buds 0.9 cm petal length	19	0.1	7.5	0.65
Disc petal stage 3 opening flowers 1.2 cm petal length	14	0.7	7.4	0.70
Disc petal stage 4 open flowers 3.5 cm diameter	11	0.5	3.5	0.70

Fisetin was purchased from Sigma–Aldrich (Vienna, Austria). Isoliquiritigenin, butein, sulfuretin, marein and maritimein were obtained from Extrasynthesis (Genay, France). Okanin and maritimetin were prepared as described previously [11].

### 2.2 Estimation of yellow pigments and enzyme preparation from *C. grandiflora* petals

0.3 g petals were homogenized in 1 ml methanol. Solid remnants were removed by centrifugation for 10 min at 13,000×*g*. Using a calibration curve obtained with sulfuretin [11], the content of yellow pigments was estimated based on the absorption at 395 nm of the supernatant and expressed as mg sulfuretin equivalents per g fresh petal weight. Methanolic extracts were subjected to enzymatic hydrolysis and HPLC analysis as previously described to obtain aurone to chalcone ratios [11].

### 2.3 Aurone synthase (AUS) activity assay

**Fig. 1:**
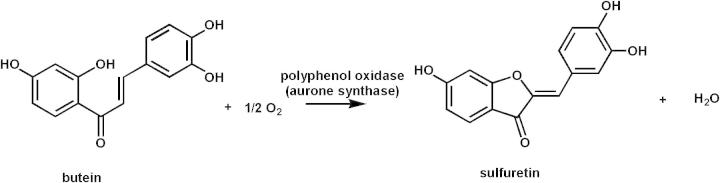
Reaction catalyzed by aurone synthase (AUS).

**Fig. 2:**
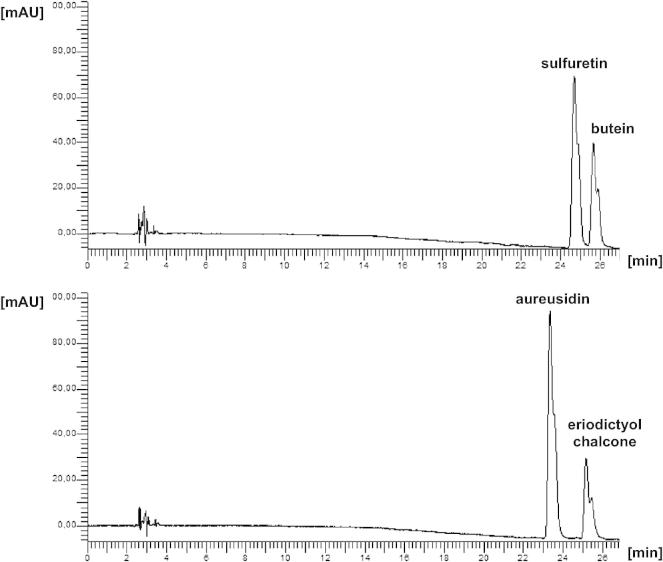
HPLC chromatograms of purified AUS1 after incubation for 15 min at 30 °C with butein (above) and eriodictyol chalcone (below) as methanolic extracts.

Enzyme preparations from petals of *C. grandiflora* and AUS assays were performed as described previously (Fig. 1) [11]. For testing the highly purified recombinant enzyme, the assay was slightly adapted from [11] by using 0.1 M K_2_HPO_4_/KH_2_PO_4_ buffer pH 5.5 and only 14 ng AUS (Fig. 2). For rapid testing during purification, a spectrophotometric activity test was applied. The reaction was performed in a 1 cm quartz cuvette using 1 ml 125 mM sodium citrate, pH 5.4 supplemented with 2.5 mM SDS as an activating agent, 75 μM fisetin and 1 μl of solution containing AUS1 at 25 °C [12]. The change in absorbance at 280 nm was monitored over time and the velocity of the reaction was determined from the initial linear portion of the curve.

### 2.4 Cloning and sequencing of cgAUS from *C. grandiflora*

Extraction of total RNA, reverse transcription and cDNA synthesis were performed using RNeasy Plant Mini Kit (Qiagen, Hilden, Germany) and SMARTer™ RACE cDNA Amplification Kit (Clontech, Saint-Germain-en-Laye, France). cDNA between the two copper atoms was amplified using Phusion Hot Start High-Fidelity DNA Polymerase (NEB, Ipswich, England) and primers 009 and 011. The obtained cDNA fragments were ligated into the vector pCR2.1-TOPO (Invitrogen, Paisley, UK), and sequenced by a commercial supplier. Full-length cDNA (without the transit peptide) was obtained using HotStarTaq Plus DNA Polymerase Kit (Qiagen) and primers 063 and 065. The PCR product was directly cloned using pTrcHis2 TOPO® TA Expression Kit (Invitrogen) with *Escherichia coli* TOP10 chemically competent cells according to the manual instruction.

### 2.5 Quantitative gene expression studies of cgAUS1 and cgAUS2

Gene expression studies were performed as previously described [13]. The analysis was carried out on three independent experiments. The data were normalized against the housekeeping genes, *elongation factor α* (*EFα*) and *actin*. Primers used for qPCR.

### 2.6 Heterologous expression and purification of recombinant AUS1 in *E. coli*

The *cgAUS1* cDNA clone was inoculated with overnight culture, grown at 37 °C in SB culture medium (3.2% tryptone, 2% yeast extract, 0.5% NaCl, supplemented with 100 μg/ml ampicillin) and induced at an OD_600_ of 0.6 with 1 mM isopropyl-β-d-thiogalactopyranosid (IPTG) and 1 mM CuCl_2_. Once the OD_600_ reached ∼1.5 the cells were harvested and washed three times in 30 mM Tris-HCl, pH 8.5 by centrifugation at 10,000×*g* for 10 min.

The pellet was resuspended in 30 mM Tris–HCl, pH 8.5 and lysed by three freeze-thaw cycles and 0.3 mg/ml lysozyme (L6876, Sigma–Aldrich). After adding 0.05 mg/ml DNaseI (Sigma–Aldrich) and 10 mM MgCl_2_ the lysate was centrifuged (25,000×*g*, 30 min, 4 °C) and protein precipitation was achieved by adding ammonium sulfate to 80% saturation. The precipitate was separated by centrifugation at 25,000×*g* for 30 min at 4 °C. The protein pellet was dissolved and purified as described in Molitor et al. (2014, submitted to FEBS).

### 2.7 Denaturing and partially denaturing SDS–PAGE analysis of AUS1

Electrophoresis was performed by the method of Laemmli [14]. The 8% polyacrylamide gels were run in a Mini-PROTEAN Tetra Cell System (BioRad, Vienna, Austria) at a constant current of 120 mV. Samples mixed with reduced loading buffer were heated for 10 min and loaded to the gel. Precision Plus Protein Standard Dual Color (Bio-Rad) was used as the molecular weight marker exhibiting a molecular mass range from 10 to 250 kDa. SDS–PAGE was stained with Coomassie Brilliant Blue R250.

In-gel AUS activity was determined by applying a partially denaturing 8% SDS–PAGE according to Cabanes et al. [15]. Samples were mixed with loading buffer (absence of *β*-mercaptoethanol) and applied without heating the samples. The gel was soaked in 125 mM sodium citrate pH 5.4 containing 41 μg/ml butein for activity staining. Formation of sulfuretin was monitored by a Typhoon 8600 (GE Healthcare, Munich, Germany) in the fluorescence mode using green laser (532 nm) for excitation and 555BP20 as emission filter.

### 2.8 Tryptic digestion and peptide mass fingerprint of AUS1

14 μg purified AUS1 enzyme in 20 μl 100 mM ammonium carbonate buffer pH 8.0 were mixed with 5 μl 100 mM ammonium carbonate buffer pH 8.0 containing 10 mM dithiothreitol (DTT) and incubated at 37 °C for 45 min. 20 μl 55 mM iodoacetamide in 100 mM ammonium carbonate buffer pH 8.0 were added and incubated for 45 min at room temperature in the dark. After adding 1 μl 0.5 mg/ml trypsin solution the reaction mixture was incubated at 37 °C over night. The digest was stopped with 0.1% trifluoroacetic acid (TFA) followed by desalting using a Ziptip C 18 (Millipore, USA) according to the manufacturer’s protocol. The solvent was removed in the vacuum.

Tryptic peptides were analyzed by using the nanoflow UPLC Ultimate 3000 RSLCnano System (Thermo Fisher Scientific) directly coupled to a LTQ Orbitrap Velos mass spectrometer (Thermo Fisher Scientific) equipped with a Nanospray Flex Ion Source (Thermo Scientific). Prior to nanoUHPLC–ESI-MS/MS measurements the lyophilized samples were diluted in 2% (v/v) acetonitrile, 0.1% (v/v) formic acid to a final concentration of 1 fmol/μL. Sample introduction (10 fmol AUS1/10 μl) was done using an RSLC nano autosampler (Thermo Scientific).

Reversed-phase chromatography included a trapping column (Acclaim PepMap 100, C18, 100 μm × 20 mm, 5 μm) and a separation column (Acclaim PepMap RSLC, C18, 75 μm × 150 mm, 2 μm) and was operated at a flow rate of 300 nl/min. The mobile phases for LC separation were 0.1% (v/v) formic acid in LC/MS grade water (solvent A) and 0.08% (v/v) formic acid in 80% (v/v) acetonitrile (solvent B). A gradient from 2% to 40% solvent B was used over 30 min following subsequent washing with 80% solvent B and preconditioning of the column to 2% solvent B prior to the next injection. The MS1 (first device in tandem mass spectrometry) scan, data acquisition and data analysis was performed using parameters.

## 3 Results and discussion

### 3.1 Cloning and sequence analysis of cgAUS from *C. grandiflora*

**Fig. 3:**
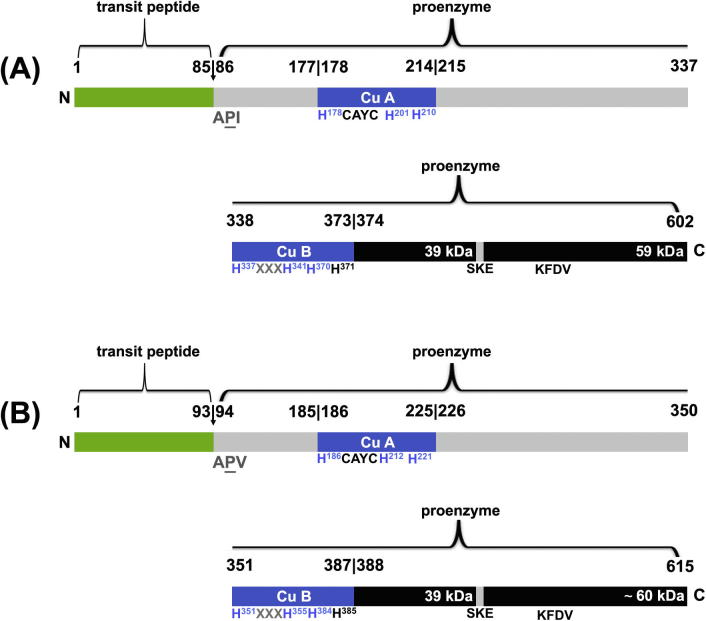
Scheme of amino acid sequence of AUS1 (A) and AUS2 (B) domains. The transit peptide is shown in green, the copper sites with the highest conserved regions and coordinating histidines in blue. The C-terminal domain is shown in black. The calculated mass of 59 kDa (AUS1) and ∼60 kDa (AUS2) is based on the pro-enzyme (core domain + C-terminal domain).

**Fig. 4:**
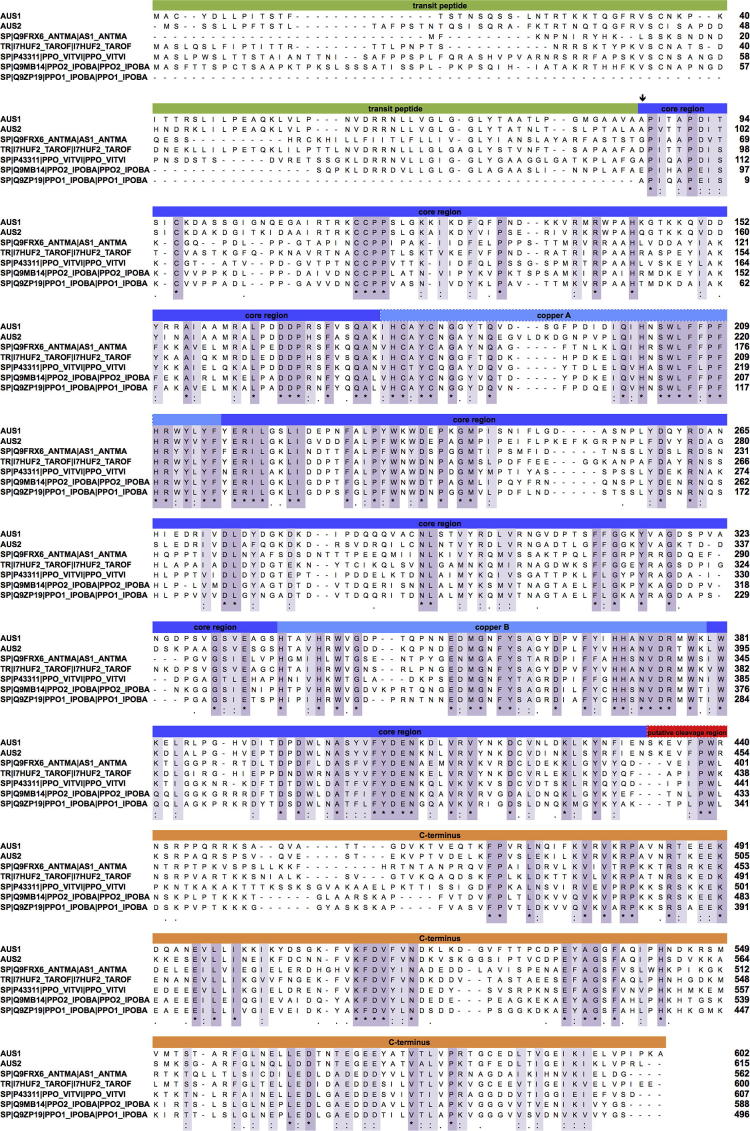
Sequence alignment of aurone synthase from *Coreopsis grandiflora* (AUS1 NCBI KC972611, AUS2 NCBI KC878307) with aureusidin synthase from *Antirrhinum majus* AmAS1 (Q9FRX6), *Taraxacum officinale* (I7HUF2), PPO from *Vitis vinifera* VvCO (P43311) and 39 kDa and 40 kDa *Ipomoea batatas* catechol oxidase IbCO (Q9MB14 and Q9ZP19).

Cloning attempts with primers based on the sequence information of aureusidin synthase *AmAS1* (NCBI AB044884) [8] failed. Partial cDNA sequences were obtained by using degenerated primers around the highly conserved copper site [16], which were completed by RACE techniques. Three cDNA clones, *cgAUS1* (KC972611), *cgAUS2a* (KC878307) and *cgAUS2b* (KC878308)*,* were isolated (Fig. 3). *cgAUS1* has an *ORF* of 1809 bp encoding for 602 amino acids, which corresponds to a mass of 68.0 kDa. The latent enzyme (also called: pro-enzyme) consists of 517 amino acids (58.9 kDa) starting with the amino acid sequence APITAPDI. The coordinating histidines of Cu_A_ are in positions 178, 201 and 210 (of the full-length amino acid sequence, including the transit peptide) and of Cu_B_ in positions 337, 341 and 370 (Fig. 4). Highly conserved regions are identified e.g. the non-copper coordinating histidine in position 371, sometimes called the seventh histidine [16], [17], [18], the HCAYC motif at the beginning of the Cu_A_ domain, the HxxxH motif in the Cu_B_ domain, and the KFDV motif in positions 512–515 in the C-terminal region [3], [17].

*cgAUS2a* and *cgAUS2b* show an ORF of 1851 bp and encode for 615 amino acids (69.2 kDa). *cgAUS2a* and *cgAUS2b* show a sequence identity of 99% with exchanges at amino acid positions 43 (isoleucin instead of asparagine in *2b*), 112 (threonin instead of isoleucin in *2b*) and 555 (arginine instead of glutamine in *2b*). They are assumed to be allelic variants and the *cgAUS2b* sequence is therefore not shown separately. The AUS2 pro-enzymes start with amino acid sequence APVTTPDI and consist of 522 amino acids corresponding to a mass of 59.1 kDa. The coordinating histidines of AUS2a/b are in positions 186, 212 and 221 for Cu_A_ and in positions 351, 355 and 384 for Cu_B_ (Fig. 3). Sequence identities between the deduced amino acid sequences of AUS1 and AUS2 are 68% (Table 2).

**Table 2:** Percentages of sequence identities of full-length clones, pro-enzyme, core region (sequence without transit peptide region and without the C-terminal region (in AUS1 from A^86^PI to S^433^KE) and transit peptide. In yellow are the sequence identities of AUS1 compared with AUS2; in orange are the sequence identities of AUS1 and AUS2 compared with AmAS1; in green are the sequence identities of AUS1 and AUS2 compared with PPO-6 from *Taraxacum offinicale*, which is most related to AUS1 and AUS2; in blue are the sequence identities of AmAS1 compared with tyrosinase from *Juglans regia*.

### 3.2 Alignments and phylogenetic tree of AUS from *C. grandiflora* with other PPOs

The obtained sequences were compared with PPOs from *A. majus* (Uniprot Q9FRX6, an aureusidin synthase), *Taraxacum officinale* (Uniprot I7HUF2, dandelion PPO), *Vitis vinifera* (Uniprot P43311, catechol oxidase), *Ipomoea batatas* (Uniprot Q9ZP19 and Q9MB14, 39 kDa and 40 kDa catechol oxidase) and *Juglans regia* (Uniprot COLU17, tyrosinase) (Fig. 4, Table 2) Both AUS pro-enzymes show highest identities to PPO-6 from *T. officinalis*, with general higher sequence identities to catechol oxidases from various plant sources, than to aureusidin synthase. In contrast, aureusidin synthase shows highest sequence identity to tyrosinase from *J. regia* [19].

Cleavage during proteolytic activation, behind the SKE motif (S^348^ and S^354^ of the pro-enzyme AUS1 and AUS2), result in a molecular mass of approximately 40 kDa, for the active enzyme. Putative cleavage sites are further discussed in Molitor et al. (2014, submitted to FEBS). The motif SKE is also present in PPO-6 of *T. officinalis* [17] at position S^398^. In catechol oxidase from *V. vinifera* (Uniprot P43311) a SK motif is present at position S^353^ which would result in a calculated molecular mass of 39.9 kDa for the active enzyme, although the molecular mass of the crystal structure showed only 38.4 kDa, ending after WLPKNTK in the putative cleavage region, leading to the reduced mass, as shown in Fig. 4 [18]. A SK motif is also present in the two isoenzymes of *I. batatas* (Uniprot Q9MB14 and Uniprot Q9ZP19) in positions S^347^ and S^343^, which lead to molecular masses of 40.0 kDa and 38.5 kDa, respectively. Molecular masses of 40.2 kDa and 38.8 kDa of these proteins were determined by MALDI–MS [20].

Knowledge about localization of the flavonoid biosynthesis, their substrates and products is still limited. However chalcone synthase, the first enzyme of the flavonoid pathway, was found to be localized in the cytosol and the endoplasmic reticulum [21]. Other investigations found co-localized chalcone synthase in addition within the nucleus of Arabidopsis roots [22]. Studies of chalcone synthase in developing as well as ripe wine grapes presented a predominant localization in the plastid (chloroplast and chromoplast), the rough endoplasmic reticulum, the cytoplasm, the vacuole and the cell wall [23]. In later stages chalcone synthase was still largely found in the plastid and the endoplasmic reticulum [23]. In this work transit peptides of various plant PPOs were analyzed using TargetP 1.1 (http://www.cbs.dtu.dk/services/TargetP/) and ChloroP 1.1 (http://www.cbs.dtu.dk/services/ChloroP/). All PPOs are predicted to contain a N-terminal cTP (chloroplast transit peptide) except for the PPO from *Populus trichocarpa* (Uniprot F8V190) [3] and aureusidin synthase from *A. majus* (Uniprot Q9FRX6) [8] which are expected to be localized in vacuoles. The diversity of the predicted locations and the differences in the main sequences suggest that biosynthesis of 4-hydroxyaurones and 4-deoxyaurones follow different pathways in different locations of the plant cell.

**Fig. 5:**
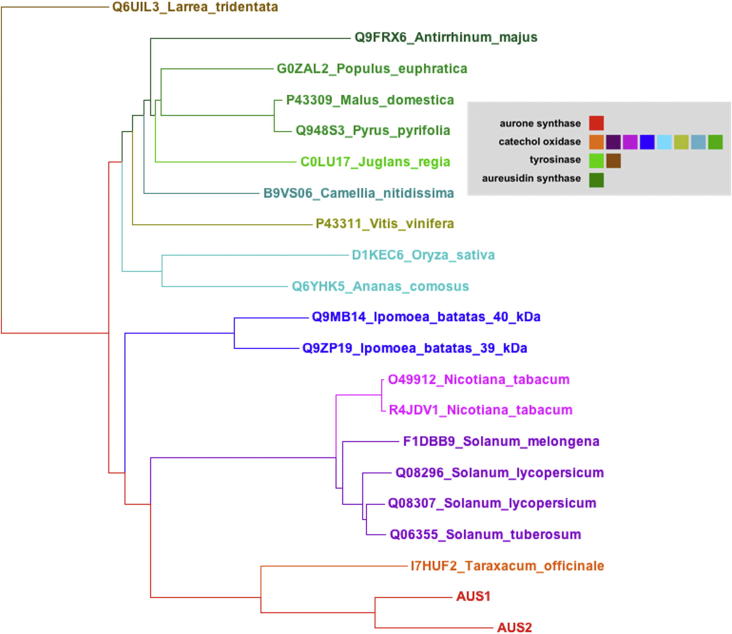
Phylogenetic tree of various plant PPOs (tyrosinases and catechol oxidases as specified in Uniprot, the first six characters in the labels at the figure are the Uniprot database accession numbers) constructed by Archaeopteryx: visualization, analysis, and editing of phylogenetic trees [24]. The length of the section indicates the relative distances between the sequences.

A phylogenetic tree of various plant PPOs was constructed with Archaeopteryx [24]. This tree obviously forms three major clusters, one containing catechol oxidases, one tyrosinase as well as aureusidin synthase. The second cluster contains only catechol oxidases. (+)-Larreatricin hydroxylase, an enantio-specific PPO from the creosote bush *Larrea tridentata* forms a third stand-alone cluster [25]. The second cluster of only catechol oxidases, forms three separate clades, one with catechol oxidases from *I. batatas*, one with catechol oxidases from *Nicotiana tabacum* and *Solanum sp.* and the third clade consists of AUS1, AUS2 and the dandelion PPO-6 from *T. officinale* (Fig. 5). This clade is a novel, uncharacterized class of plant PPOs, with specific functions, as described also in Dirks-Hofmeister et al. [26]. In contrast aureusidin synthase from *A. majus* has a closer relationship to plant PPOs that perform monophenolase activity as tyrosinase from *J. regia* [19], [27].

### 3.3 Quantitative gene expression studies

**Fig. 6:**
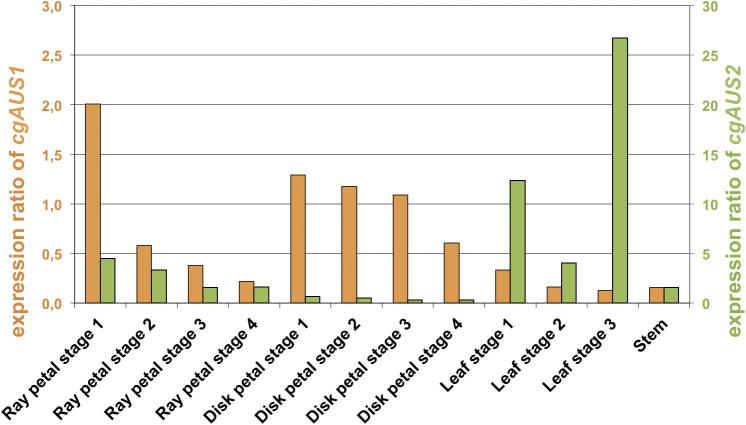
Expression ratio of *cgAUS1* (orange) and *cgAUS2a* (green) in different tissues of *Coreopsis grandiflora* normalized to *EF α*. Note the different scales of the *y*-axis for *cgAUS1* and *cgAUS2a*.

Transcription rates of the genes *cgAUS1* and *cgAUS2a*/*cgAUS2b* were quantified in different tissues including stems and developmental stages of petals and leaves (Fig. 6). *Ribulose-1,5-bisphosphate carboxylase*/*oxygenase* (*RuBisCO)*, *actin* and *EFα* were tested as housekeeping genes for normalization. *RuBisCO* turned out to be unsuitable due to an enhanced expression in leaves, the main photosynthetically active tissue. The expression of *actin* and *EFα* in contrast, was more stable throughout stages and tissues and provided consistent information (Fig. 6). *cgAUS2* shows a tenfold higher expression and exhibits highest expression rates in the leaves. *cgAUS1* shows higher expression levels in petals (Fig. 6) compared to leaves with a decreasing expression during flower development, which correlates with the decreasing aurone concentrations over time (Table 1). Yellow pigmentation of *C. grandiflora* is caused by chalcones, aurones and carotenoids, therefore particularly the last column (aurone/chalcone ratio) indicates in which developmental stages aurone formation occurs. This suggests a particular physiological relevance of *cgAUS1* in flowers and supports the assumption that *cgAUS1* is particularly involved in pigment formation, whereas *cgAUS2* is a common PPO with no specific role in pigment formation. The involvement of *cgAUS1* in 4-deoxyaurone formation was furthermore confirmed by purification of AUS activity from *C. grandiflora* flowers to homogeneity, amino acid sequence determination and comparison with the cDNA sequence of the here presented *cgAUS2* cDNA clone (Molitor et al. submitted to FEBS). Heterologous expression therefore focused on AUS1.

### 3.4 Heterologous expression, purification and identification of AUS1

**Fig. 7:**
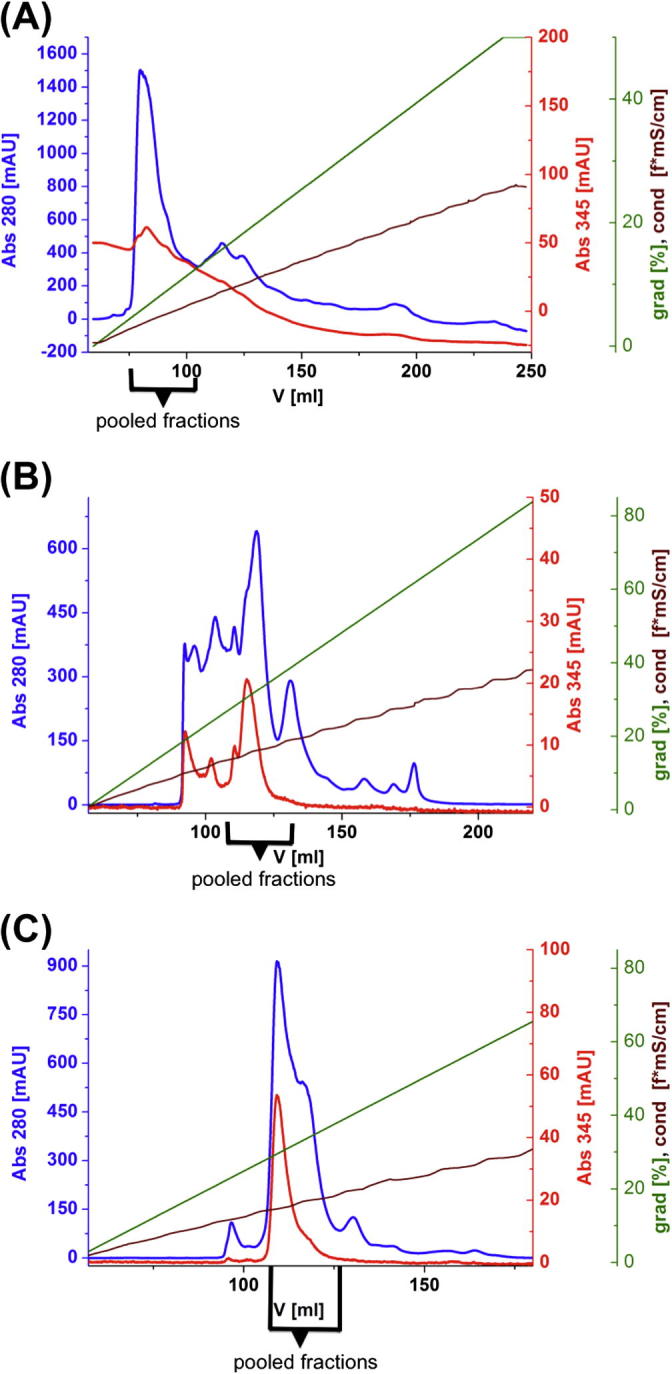
Chromatographic (FPLC) runs. (A) Anion exchange chromatography on MonoQ, (B) cation exchange chromatography on MonoS and (C) cation exchange chromatography on MonoS. Legend: UV absorbance at 280 nm [mAU], UV absorbance at 345 nm [mAU], gradient [% buffer B], conductivity [mS/cm] (*f* = ∼0.5).

**Fig. 8:**
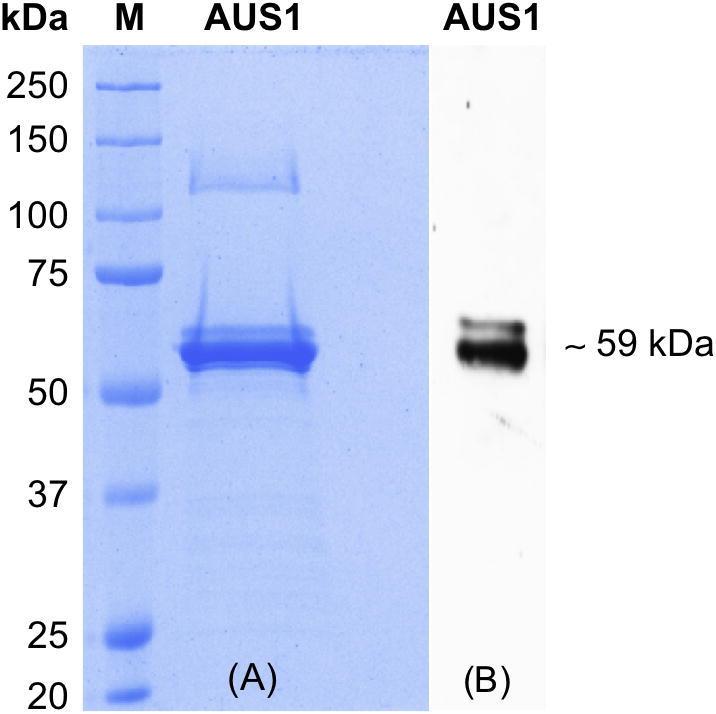
Recombinant, purified AUS1 analyzed by 8% SDS–PAGE. (A) Sample was reduced for 10 min by adding 1 mM DTT and gel was stained with Coomassie and (B) AUS1 was untreated and gel was stained with butein and sulfuretin formation monitored by a Typhoon 8600 (green laser, emission filter 555BP20).

Heterologous expression in *E. coli* of the cDNA clone encoding the pro-enzyme resulted in the formation of 5–6 mg soluble native holo-pro (latent) AUS1 per liter of culture medium. As expression yielded an inactive enzyme while incorporating a His-tag (an additional N- or C-terminal motif consisting of six histidine residues), the enzyme was thus expressed without any tag. Recombinant AUS1 was purified to homogeneity by four subsequent chromatographic steps, including one cation exchange column (SP-Sepharose FF), one anion exchange column MonoQ (Fig. 7A) and another cation exchange column MonoS twice under identical conditions (Fig. 7B and C). After each chromatographic step, the collected fractions were tested for latent AUS1 (pro-enzyme) activity, using SDS as activating agent, due to binding of SDS inducing the activation of latent AUS1 [28]. Active fractions were pooled and subjected to the next ion exchange column. Homogeneity of recombinant AUS1 was proven by SDS-PAGE, which shows a single band of approx. 59 kDa (Fig. 8A). AUS activity of this band was demonstrated via staining a partially denaturing 8% SDS-PAGE with butein (Fig. 8B).

Tryptic peptides of the AUS1 amino acid sequence were identified by nanoUHPLC–ESI-MS/MS. In total, fragments that spanned a 428 amino acid segment are verified, which represent 78% of the protein encoding for the 59 kDa pro-enzyme (accession number KC972611). This confirms that the gene sequence belongs to the purified recombinant enzyme. The first peptide starts with the sequence ALAPI missing the methionine, which was already removed due to posttranslational processing by the expression system *E. coli*. The sequence of the amino acids forming the putative thioether bridge (C97 to H116 coordinated to Cu_A_ in the pro-enzyme) was not found, most likely due to the large mass of 4.1 kDa, resulting on the sequence of IHCAYCNGGYTQVDSGFPDIDIQIHNSWLFFPFHR. Such a big peptide might have a low grade of ionization and be therefore hard to detect.

### 3.5 Aurone synthase activity and presence of yellow pigments in *C. grandiflora*

Aurone synthase activity and aurone concentrations were determined during flower development. The yellow flower color of *Bidens ferulifolia* [11] and *C. grandiflora* is a result of the accumulation of carotenoids and deoxyanthochlors, particularly of derivatives of butein, sulfuretin, okanin and maritimetin [7]. Incubation of butein with enzyme preparations from *C. grandiflora* petals resulted in the formation of a single product, which was identified as sulfuretin, according to Miosic et al. [11] (Fig. 1). No product formation was observed when isoliquiritigenin (one hydroxyl group in the B-ring) was used as a substrate, suggesting that in *C. grandiflora* a catechol oxidase homolog is responsible for aurone formation. When NADPH was added to the assay, formation of butein and sulfuretin (both two hydroxyl groups in the B-ring) from isoliquiritigenin could be observed. This suggests the involvement of a cytochrome P450 dependent monooxygenase in the introduction of an additional hydroxyl group in the B-ring. Thus, in contrast to *A. majus*, hydroxylation and 4-deoxyaurone formation in *C. grandiflora* is catalyzed by two separate enzymes, chalcone 3-hydroxylase and aurone synthase, as described for *B. ferulifolia* [11].

Compared to ray petals, disk petals show lower concentrations of yellow pigments and there was a continuous decrease of pigment concentrations (aurones, chalcones, and carotenoids) during flower development. Highest concentrations of aurones (aurone:chalcone ratio in the last column in Table 1) are observed in closed buds, but the aurone:chalcone ratio strongly decreases in the later stages and is highest (0.41) in the earliest stages, ray petal stage 1 and 2 (closed buds) (Table 1). Therefore, young petals of *C. grandiflora* were chosen as source for the isolation of an *cgAUS* cDNA clone. In contrast, highest AUS activities per g fresh weight is found in opening flowers (petal stage 3) and highest specific activity in open flowers (petal stage 4). The pigment level decreases during later developmental stages, whereas the activity increases until petal stage 3. This might be explained by a putative bifunctional role of AUS. It was shown that AUS is also capable to oxidize several other diphenols, such as fisetin. Therefore an additional role of AUS1, beside aurone formation, might be possible. The high AUS activity in late developmental stages might also be due to the presence of other PPOs, like AUS2a and AUS2b, which are highly expressed in leafs, but also found in petals.

The purified recombinant enzyme AUS1 was tested in vitro with different substrates, as chalcones and flavonols. The flavonol fisetin is also accepted as described for the PPO from *Vicia faba* [12]. Incubation of butein with AUS results only in the formation of sulfuretin due to oxidative cyclization (Fig. 1, Fig. 2). When eriodictyol chalcone was used as a substrate, formation of aureusidin but no bracteatin, was detected (Fig. 2). The specific activity is 6 kat/kg AUS1 with both butein and eriodictyol chalcone as substrates. Naringenin chalcone or isoliquiritigenin, which possess only one hydroxyl group in position 4, however was not converted. This is in contrast to aureusidin synthase which shows a preference for dihydroxylated chalcones in ring B, but accepts monohydroxylated substrates as well when hydrogen peroxide is present [8]. Kinetic data (Molitor et al., 2014 (submitted to FEBS) have been recorded on the enzyme purified from natural source, which comes the *in vivo* situation closest. The results presented in this paper suggest that formation of 4-deoxyaurones in Asteraceae species follows a different mechanism than 4-hydroxyaurone formation in *A. majus* on a molecular level. Although a cDNA clone was isolated from *A. majus* encoding aureusidin synthase [8], heterologous expression of the enzyme has not been demonstrated so far. Thus, this work reports on the first successful heterologous expression of an enzyme involved in aurone formation. This lays the foundation for the production of high amounts of pure protein for crystallization attempts and also allows the generation of mutated AUS for structure-activity studies.

## Conflict of interest

The authors declare no competing financial interest.
