# Supplementary material for: Cloning and functional expression in E. coli of a polyphenol oxidase transcript from Coreopsis grandiflora involved in aurone formation
Source: FEBS Lett. 2014 Sep 17;588(18):3417–26. doi: 10.1016/j.febslet.2014.07.034 (PMC4158910; doi:10.1016/j.febslet.2014.07.034)

**Cloning and functional expression in *E. coli* of a polyphenol oxidase transcript from *Coreopsis grandiflora* involved in aurone formation**

Cornelia Kaintz^1^, Christian Molitor^1^, Jana Thill^2^, Ioannis Kampatsikas^1,2^, Claudia Michael^3^, Heidi Halbwirth^2^, Annette Rompel^1^

^1^Universität Wien, Fakultät für Chemie, Institut für Biophysikalische Chemie, Althanstraße 14, 1090 Wien, Austria

^2^University of Technology Vienna, Institute of Chemical Engineering, Getreidemarkt 9, 1060 Vienna, Austria

^3^University of Vienna, Department of Analytical Chemistry, Währinger Straße 38, 1090 Vienna, Austria

To whom correspondence should be addressed:

Annette Rompel

phone: +43 1 4277 52502

fax: +43 1 4277 9525

email: annette.rompel@univie.ac.at

Figure S 1 A and B: Tyrosinase catalyzes the conversion of monophenols to *o*-quinones via *o*-diphenols. B: Catechol oxidase catalyzes only the second step (*o*-diphenol to *o*-quinone).


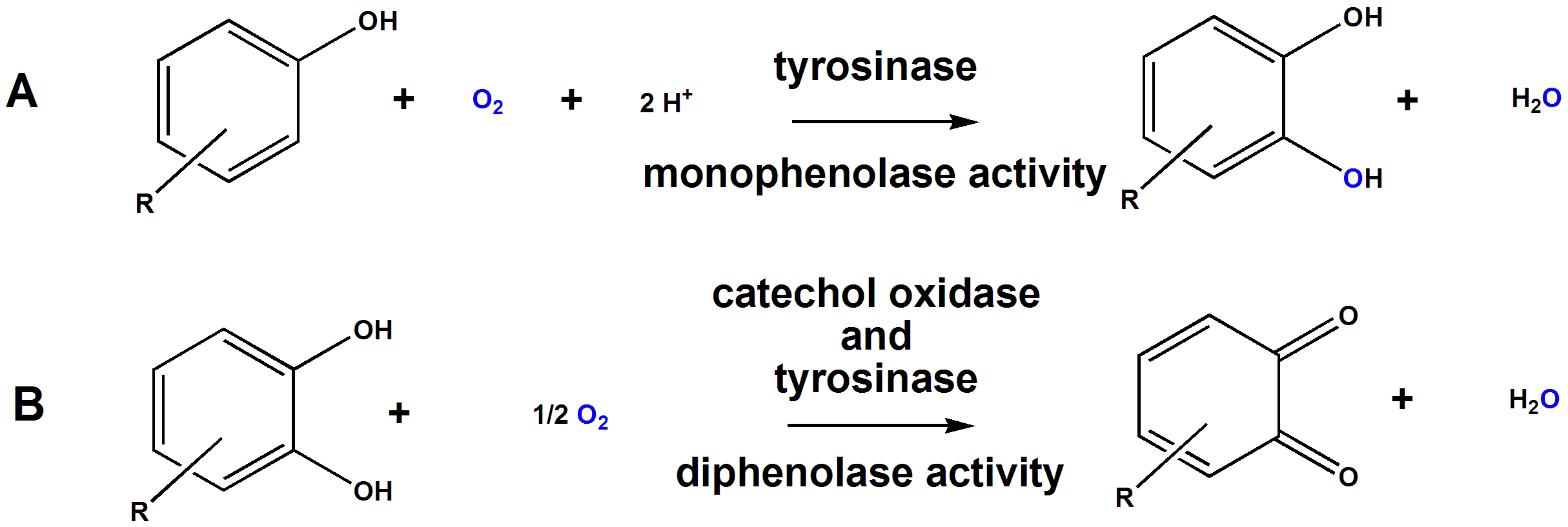


**Figure S 2 Structures of chalcones, aurones and flavonols.**


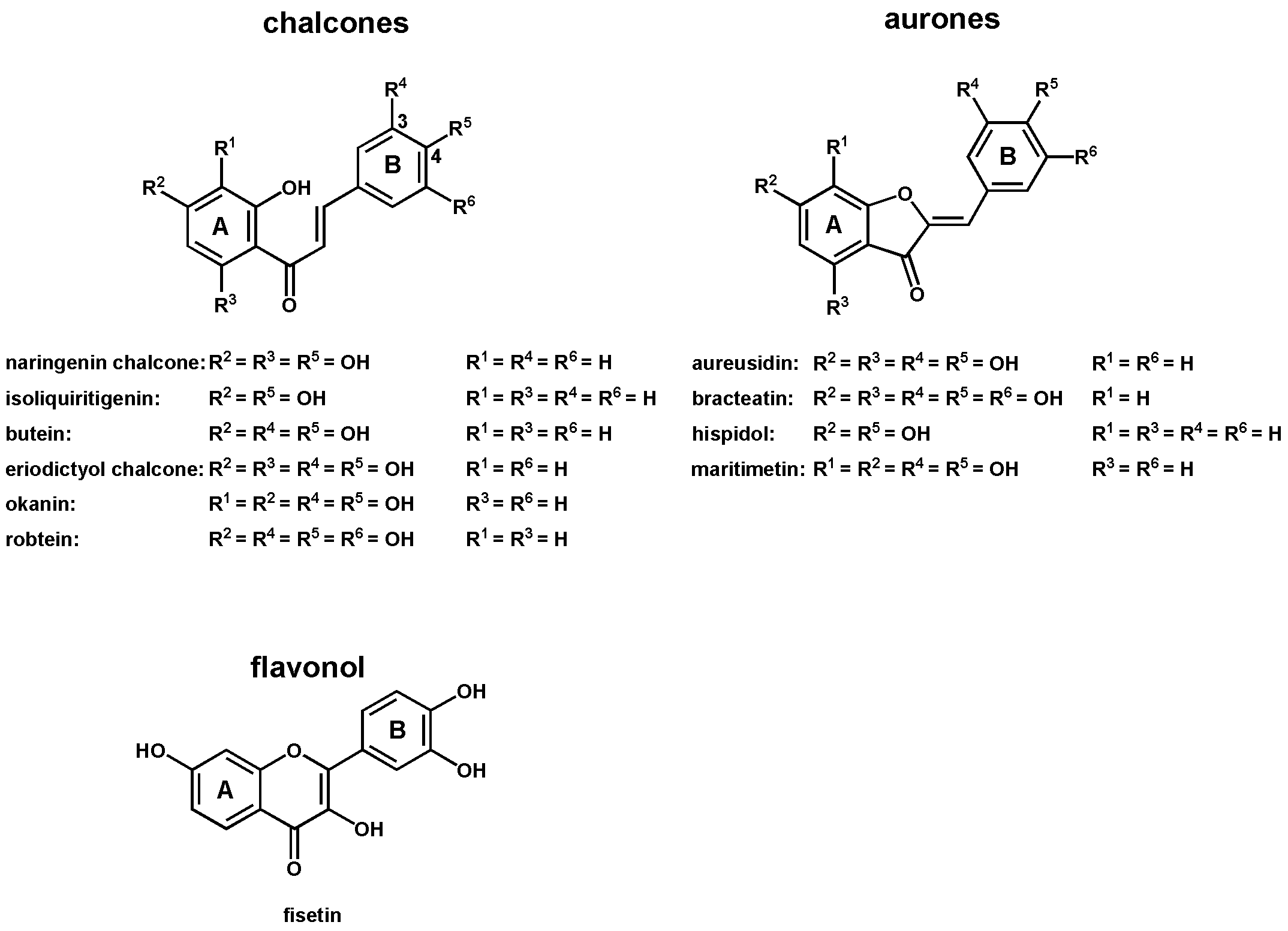


**Figure S 3 HPLC chromatograms from incubation of a, b: protein preparations from petals of *C. grandiflora* with isoliquiritigenin in the absence (a) and presence (b) of NADPH.
c-g: purified AUS1 with isoliquiritgenin (c), THC (d), robtein (e), okanin (f) and marein (g)
abbrev.: iso: isoliquiritigenin, but: butein, sul: sulfuretin, THC: 2’,4’,6’,4-tetrahydroxychalcone, THA: 3’,4’,5’,6-tetrahydroxyaurone, HPLC chromatography was performed according to a-d:** [**Vande Casteele, et al.**](#_ENREF_36) **[29], e-f:** [**Chandra, et al.**](#_ENREF_3) **[30].**


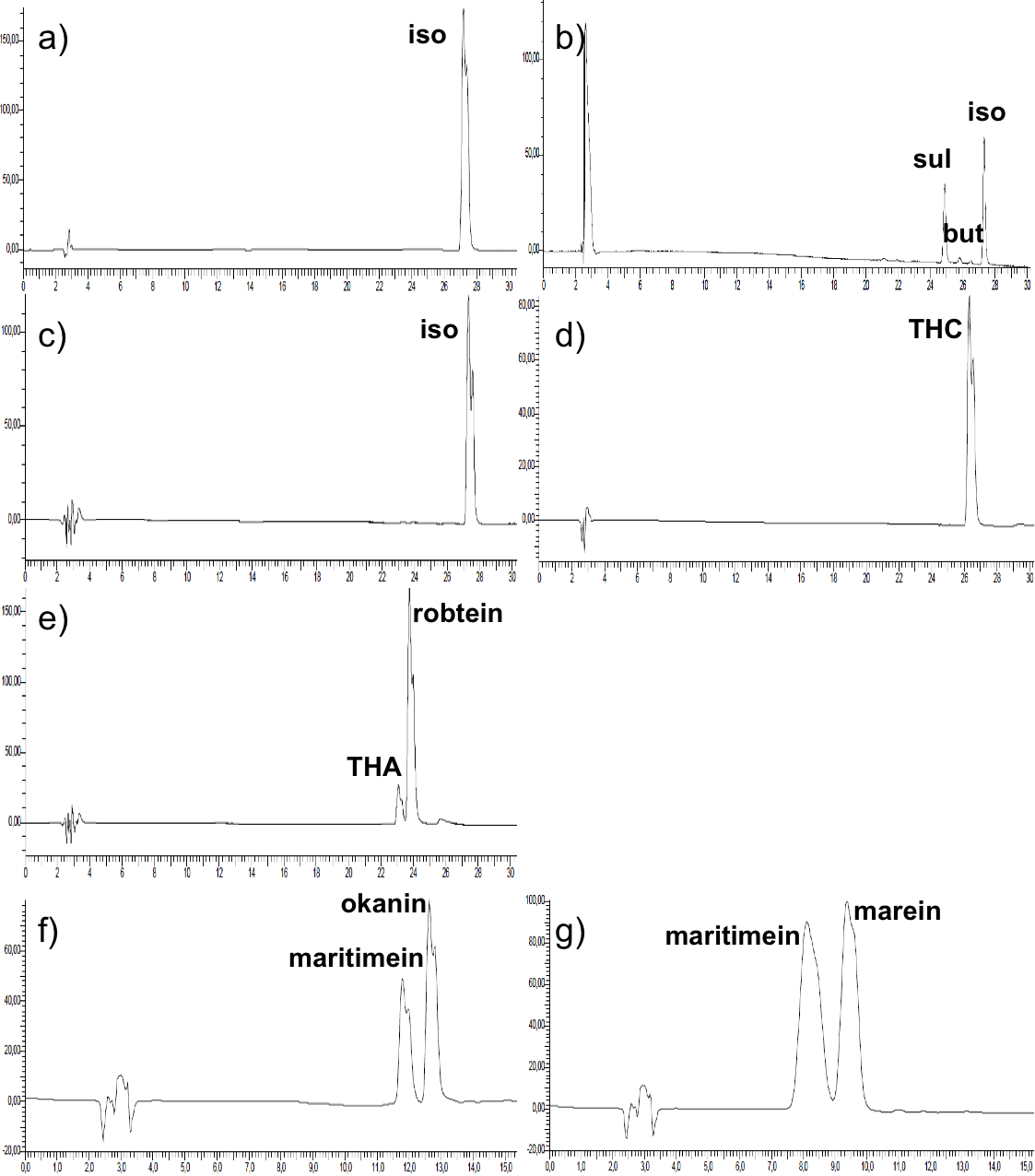


[29] Vande Casteele, K., Geiger, H. and Van Sumere, C.F. (1982) Separation of flavonoids by reversed-phase high-performance liquid chromatography. J. Chromatogr. A 240, 81–94.

[30] Chandra, A., Rana, J. and Li, Y. (2001) Separation, identification, quantification, andmethod validation of anthocyanins in botanical supplement raw materials by HPLC and HPLC–MS. J. Agric. Food Chem. 49, 3515–3521.

Figure S 4 cDNA sequence of *cgAUS1* (NCBI accession number KC972611) from *C. grandiflora* and translated amino acid sequence. Underlined nucleotide sections are the used primers for sequencing and cloning. The beginning of the N-terminal sequence after the transit peptide is shown in green. The highly conserved regions with the copper binding sites are shown in blue with the copper coordinating histidines highlighted in yellow.

atggcgtgttatgatcttcttccaatcacctccactttcacttccactaactcccaatct 60

M A C Y D L L P I T S T F T S T N S Q S 20

tcccttaatactcgtaccaagaaaacccaagggtttagagtttcatgcaacaagcccaag 120

S L N T R T K K T Q G F R V S C N K P K 40

ataacaacaagaagtctcatactacccgaagcccaaaagcttgtactccccaatgtagac 180

I T T R S L I L P E A Q K L V L P N V D 60

cgaaggaaccttctggtsggacttggcggcctttacacggccgccaccctcccaggaatg 240

R R N L L X G L G G L Y T A A T L P G M 80

ggagcagcagtagctgctcccataacagctcctgacatcacgtccatttgcaaggacgcg 300

G A A V A A P I T A P D I T S I C K D A 100

Agttcaggaattggcaaccaggagggcgccataagaacaagaaaatgctgccctcctagc 360

S S G I G N Q E G A I R T R K C C P P S 120

Ctcggaaaaaagataaaagatttccaatttccgaatgacaagaaggtgaggatgagatgg 420

L G K K I K D F Q F P N D K K V R M R W 140

ccggcacataaagggacaaagaagcaagtcgacgattatcgaagagccatcgcagccatg 480

P A H K G T K K Q V D D Y R R A I A A M 160

cgagctctcccggatgatgatccacgtagctttgtcagccaagctaaaatccattgtgct 540

R A L P D D D P R S F V S Q A K I H C A 180

tattgtaacggtgggtacactcaagtcgatagcgggttccctgatatcgatattcagatt 600

Y C N G G Y T Q V D S G F P D I D I Q I 200

cacaactcatggcttttctttccgttccatcgctggtacctttatttctacgaaagaatc 660

H N S W L F F P F H R W Y L Y F Y E R I 220

ctcggaagcttgattgatgagccgaatttcgcattaccgtactggaaatgggacgaaccc 720

L G S L I D E P N F A L P Y W K W D E P 240

aagggaatgcccatttcgaatatcttcttgggcgatgcatcaaaccccttgtatgaccaa 780

K G M P I S N I F L G D A S N P L Y D Q 260

tacagggatgctaatcatatcgaagatcgaatcgtcgatctcgactatgatggcaaggat 840

Y R D A N H I E D R I V D L D Y D G K D 280

aaagatatcccagaccagcaacaagtagcatgcaatttgagtacggtgtaccgagacttg 900

K D I P D Q Q Q V A C N L S T V Y R D L 300

gtaagaaatggggttgaccctacaagtttctttggaggcaaatacgttgcgggggactcc 960

V R N G V D P T S F F G G K Y V A G D S 320

cccgttgcgaatggagacccgtcagtcgggtccgtggaagcgggttcccacacggccgtg 1020

P V A N G D P S V G S V E A G S H T A V 340

catagatgggtaggtgacccaacacagcctaacaacgaggacatgggaaacttctactcc 1080

H R W V G D P T Q P N N E D M G N F Y S 360

gctgggtatgaccccgtgttttatatccaccatgcgaatgtcgaccgaatgtggaagcta 1140

A G Y D P V F Y I H H A N V D R M W K L 380

tggaaggaattacgccttccgggacacgttgacataacggatccagactggttaaacgcc 1200

W K E L R L P G H V D I T D P D W L N A 400

tcatatgtgttctatgacgagaataaggatcttgtccgtgtttacaacaaggattgtgtc 1260

S Y V F Y D E N K D L V R V Y N K D C V 420

aacttggacaaactcaagtataactttattgaaaactctaaggaagtgttcccatggcgc 1320

N L D K L K Y N F I E N S K E V F P W R 440

Aacagtcgcccgccgcaacgtagaaagagtgcccaggttgcgacgactggagatgtgaag 1380

N S R P P Q R R K S A Q V A T T G D V K 460

acggtggagcaaaccaagttcccggtgcgtctaaaccagatctttaaggttcgtgtgaag 1440

T V E Q T K F P V R L N Q I F K V R V K 480

cggccggctgtgaacaggactgaagaagagaaggatcaagccaatgaggtgttgttgatc 1500

R P A V N R T E E E K D Q A N E V L L I 500

aagaaaattaagtatgatagtgggaagtttgtcaaatttgatgtgtttgtgaatgacaaa 1560

K K I K Y D S G K F V K F D V F V N D K 520

cttaaggatggtgtttttacgacgccgtgtgatcctgagtacgcgggtgggtttgcgcag 1620

L K D G V F T T P C D P E Y A G G F A Q 540

attccgcataatgataagagaagcatggttatgacgagtactgcgaggtttgggctaaac 1680

I P H N D K R S M V M T S T A R F G L N 560

gagttgttggaggacacaaataccgaaggtgaggagtatgcgacagtgacgttggtgcca 1740

E L L E D T N T E G E E Y A T V T L V P 580

aggacagggtgcgaagatctcaccgttggcgagatcaagatcgagttggttcctattcct 1800

R T G C E D L T V G E I K I E L V P I P 600

aaagcctag 1809

K A - 602

Figure S 5 cDNA sequence of *cgAUS2a* (NCBI accession number KC878307) from *C. grandiflora* and translated amino acid sequence. Underlined nucleotide sections are the used primers for sequencing and cloning. The beginning of the N-terminal sequence after the transit peptide is shown in green. The highly conserved regions with the copper binding sites are shown in blue with the copper coordinating histidines highlighted in yellow.

atgtcttcttcgctacttccttttacctcaaccctcactgcctttccttccacaaatacc 60

M S S S L L P F T S T L T A F P S T N T 20

caatccatttcccaaagagccttcaagactcgaaccaaccaaacccaaggtttcagggtc 120

Q S I S Q R A F K T R T N Q T Q G F R V 40

tcatgcaacagtgcaccagatgaccacaatgacaggaaacttatactacccgaagcacaa 180

S C N S A P D D H N D R K L I L P E A Q 60

aagcttgtactcccaaatgtagaccgaaggaacctcctcgtggggctcggcggtctctac 240

K L V L P N V D R R N L L V G L G G L Y 80

actgccaccaacctcacttcattacccacagcattagctgcgcccgtaacaactcctgat 300

T A T N L T S L P T A L A A P V T T P D 100

atcacatcgatatgcaaggacgcgaaagatgggattacgaagattgatgccgcgataagg 360

I T S I C K D A K D G I T K I D A A I R 120

acacgaaaatgttgccctcctagccttggaaaggcaattaaagactacgtgattccttca 420

T R K C C P P S L G K A I K D Y V I P S 140

gaaaggattgtgagaaagagatggccggcgcatcaagggacaaaaaagcaggtggatgat 480

E R I V R K R W P A H Q G T K K Q V D D 160

tatataaatgccatcgcagccatgagagctctcccagatgacgacccacacagctttgcg 540

Y I N A I A A M R A L P D D D P H S F A 180

agccaagcgaaaatccattgtgcttattgtaatggagcttacaatcaagaaggtgtatta 600

S Q A K I H C A Y C N G A Y N Q E G V L 200

gataaagatggtaatcctgtccctcttcagattcacaactcatggctcttctttcctttc 660

D K D G N P V P L Q I H N S W L F F P F 220

catcgatggtacgtttatttctacgagaggatactcggaaagctaattggcgttgatgat 720

H R W Y V Y F Y E R I L G K L I G V D D 240

ttcgcgttacctttctggaaatgggacgaaccagccggaatgccgattccagaaatcttt 780

F A L P F W K W D E P A G M P I P E I F 260

ctaccgaaagaatttaaaggtagaccaaatcctttgtttgatgtttaccgagatgccgga 840

L P K E F K G R P N P L F D V Y R D A G 280

agtcttgaagatagaatagtggatctggccttccaaggganagataaagatagatccgtg 900

S L E D R I V D L A F Q G X D K D R S V 300

gacaggcagatactttgtaatcttaatactgtgtaccgagatttagtcagaaacggagct 960

D R Q I L C N L N T V Y R D L V R N G A 320

gatacactaggcttctttgggggcaaatatgtcgctggaaaaactgatgacgactctaag 1020

D T L G F F G G K Y V A G K T D D D S K 340

cccgcagctggatcggtggaatccgggagtcacactgccgtgcatcgatgggtaggcgat 1080

P A A G S V E S G S H T A V H R W V G D 360

gacaaacagcctaatgatgaagacatgggtaacttttattccgcagggtatgatcctttg 1140

D K Q P N D E D M G N F Y S A G Y D P L 380

ttttatgttcatcatgctaatgttgatcgtacgtggaagctatggaaggatttagccctt 1200

F Y V H H A N V D R T W K L W K D L A L 400

ccgggacatgtggaaccaaccgatccagactggttaaacgcgtcatatgtgttctatgac 1260

P G H V E P T D P D W L N A S Y V F Y D 420

gagaataagaatctcgtacgtgtttataataaagattgcgtcgacataaacaagctcagt 1320

E N K N L V R V Y N K D C V D I N K L S 440

tatcgcttcattgaaaactccaaggaagtgttcccttggcgcaagagtcgtcccgcccaa 1380

Y R F I E N S K E V F P W R K S R P A Q 460

cgtagcccaagcgtccaagttgaatccacagaaaaggtgccaacggtggatgaattgaag 1440

R S P S V Q V E S T E K V P T V D E L K 480

ttcccagtgagtctagagaaaatcctgaaggttcgtgtgaagaggccggctgtgaatagg 1500

F P V S L E K I L K V R V K R P A V N R 500

accaaagaggagaagaagaaggagagtgaagttctactgataaatgaaataaagttcgac 1560

T K E E K K K E S E V L L I N E I K F D 520

tgcaataactttgtgaagttcgatgtgtttgtaaatgacaaggttagtaaaggcggtagt 1620

C N N F V K F D V F V N D K V S K G G S 540

atacccacggtttgtgatccggaatacgcgggttcttttgcacagattccgcatagtgat 1680

I P T V C D P E Y A G S F A Q I P H S D 560

gtgaagaaagcgtccatgaagagtggagctaggtttgggctaaatcagttgttggatgac 1740

V K K A S M K S G A R F G L N Q L L D D 580

acaaatacggaaggtgaagagtatgcaacggtggcattggttccaaagacagggtttgag 1800

T N T E G E E Y A T V A L V P K T G F E 600

gatctcaccataggcgagatcaaaattaagttggttcctcggctttaa 1851

D L T I G E I K I K L V P R L - 615

Figure S 6 Expression of *cgAUS1* (orange) and *cgAUS2* (green) in different tissues of *Coreopsis grandiflora* normalized to *actin*. Note the different scales of the y-axis for *cgAUS1* and *cgAUS2a*.


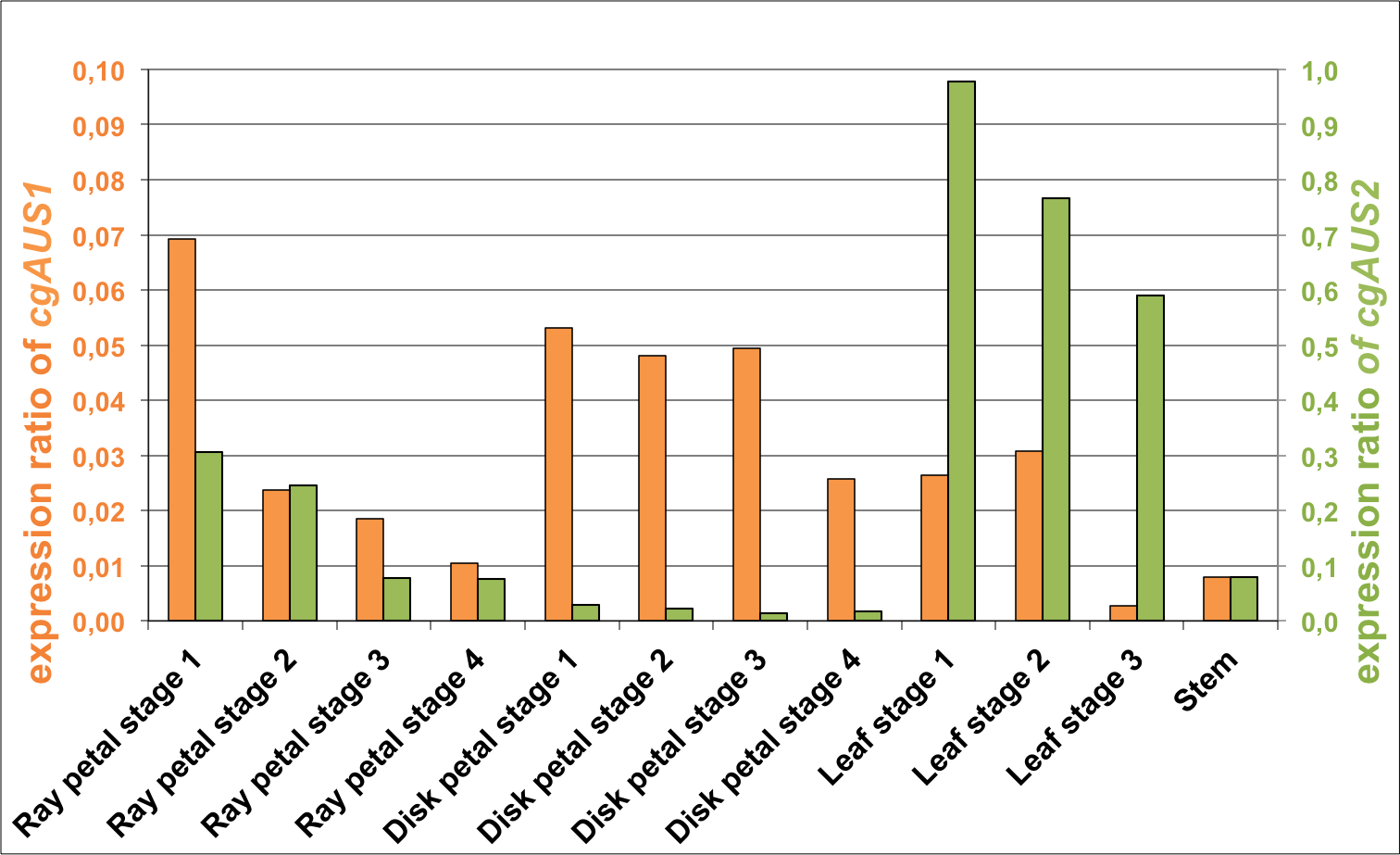

Supplement: Supplementary data 1 [file mmc1.docx]
