# Supplementary material for: Cloning and functional expression in E. coli of a polyphenol oxidase transcript from Coreopsis grandiflora involved in aurone formation
Source: FEBS Lett. 2014 Sep 17;588(18):3417–26. doi: 10.1016/j.febslet.2014.07.034 (PMC4158910; doi:10.1016/j.febslet.2014.07.034)
Supplement: Supplementary data 2 [file mmc2.docx]

**Cloning and functional expression in *E. coli* of a polyphenol oxidase transcript from *Coreopsis grandiflora* involved in aurone formation**

Cornelia Kaintz^1^, Christian Molitor^1^, Jana Thill^2^, Ioannis Kampatsikas^1,2^, Claudia Michael^3^, Heidi Halbwirth^2^, Annette Rompel^1^

^1^Universität Wien, Fakultät für Chemie, Institut für Biophysikalische Chemie, Althanstraße 14, 1090 Wien, Austria

^2^University of Technology Vienna, Institute of Chemical Engineering, Getreidemarkt 9, 1060 Vienna, Austria

^3^University of Vienna, Department of Analytical Chemistry, Währinger Straße 38, 1090 Vienna, Austria

To whom correspondence should be addressed:

Annette Rompel

phone: +43 1 4277 52502

fax: +43 1 4277 9525

email: annette.rompel@univie.ac.at

Table S 1 Primers designed for PCR amplification of *cgAUS* gene in *C. grandiflora*. The total sequence of *cgAUS* is shown in Figure S 4 and Figure S 5, where all the primers used for sequencing and cloning are underlined.

| **Primer** | **sequence (5'** → **3')** | **purpose** |
| --- | --- | --- |
| 009 forward | tggytnttyttyccnttycay | degenerated primer at copper A binding site |
| 011 reverse | catncgrtcnacrttnswrtgrtg | degenerated primer at copper B binding site |
| 018 forward | ggttcccacacggccgtgcataga | RACE-PCR for sequencing |
| 019 reverse | tttatccttgccatcatagtc | RACE-PCR for sequencing |
| 023 reverse | acaaacaacctgaccgtgatgtac | RACE-PCR for sequencing |
| 024 forward | gatgtcaggagctgttatgggagc | RACE-PCR for sequencing |
| 040 reverse | ggggagtacaagcttttgggcttcggg | RACE-PCR for sequencing |
| 041 forward | gatgtgaagacggtggagcaaaccaag | RACE-PCR for sequencing |
| 063 forward | gctcccataacagctcctgacatc | amplification of full-length cDNA |
| 065 reverse | ctaggctttaggaataggaacc | amplification of full-length cDNA |
| Anchor | cacgcgtatcgatgtcgac | RACE-PCR for sequencing |
| AUS1 forward | ggacccttcttcatttctaaactcattatg | amplification of full-length cDNA MA048 |
| AUS1 reverse | ttaaagccgaggaaccaacttaa | amplification of full-length cDNA MA048 |
| AUS2q forward | tcccagaccagcaacaagtag | Realtime PCR |
| AUS2q reverse | ccattcgcaacgggggag | Realtime PCR |
| AUS1q forward | gtccaagttgaatccacagaaaag | Realtime PCR |
| AUS1q reverse | cagtagaacttcactctccttctt | Realtime PCR |
| Actq forward | gattctggagatggtgtgtcac | Realtime PCR |
| Actq reverse | tgaataccagcagcttccat | Realtime PCR |
| EFαq forward | caaatgatctgttgttgtaacaagatatg | Realtime PCR |
| EFαq reverse | ccttgtaccagtcaaggttggt | Realtime PCR |

Table S 2 Experimental settings for nanoUHPLC-ESI-MS/MS measurements and data evaluation. Sample: Tryptic digest of purified enzyme (AUS1, 59 kDa).

| **Reduced sample lTYR; run 1 (measured by the Department for Analytical Chemistry)** | |
| --- | --- |
| Device and operating software used | Nano-HPLC (*Dionex Corporation*), Chromeleon Client Version 6.80 (*Dionex Corporation*); LTQ Orbitrap Velos (*Thermo Scientific*), LTQ Tune Plus Version 2.6.0 1065 SP3 (*Thermo Scientific*) |
| Software used (peak list generating) | Xcalibur 2.2 SP1.48 (*Thermo Scientific*) |
| Acquisition parameters | MS1 scan: m/z 400 – 1400; Filling time: 500 ms with 10^6^ ions; Resolution: 60.000; Fragmentation: CID with 35 eV; Peak picking: Top10 (intensity) with isolation window 3 m/z; Resolution; 7.500; Target ion previously selected for fragmentation were dynamically excluded for 180 s with relative mass window of 5 ppm. |
| **Search Parameters** | |
| Search engine | Peaks studio 6.0 |
| Enzyme specify | Trypsin |
| Number of miss cleavages permitted | 3 |
| Number of non-specific cleavage | 1 |
| Fixed modifications (including residue specificity) | Carbamidomethyl (cysteine) / +57.02 Da |
| Variable modifications (including residue specificity) | Oxidation (methionine) / +15.99 Da |
| Mass tolerance for precursor ions | ±5 ppm |
| Mass tolerance for fragment ions | ±0.5 Dalton |
| Name of database searched (version/date) | UniProt (UniProtKB/Swiss-Prot, March 2013); sequence of pro-aurone synthase 1 added manually |
| Species restriction and justification for searching only a subset of a database | no |
| Number of protein entries in the database actually searched | 35502518 |
| Cut-off score/expectation value for accepting individual MS/MS spectra provided | -10lgP ≥ 15 |
| Software used for PTM determination | Peaks Studio 6.0 |

Table S 3 Parameter MIAPE (Minimum Information about a Proteomics Experiment).

| Classification | Definition |
| --- | --- |
| **1. Ion sources — 1.1 Electrospray Ionisation (ESI)** |  |
| Supply type (static, or fed) | fed by nano-HPLC via |
| Interface manufacturer, model | Nanospray Flex Ion Source (Thermo Scientific) |
| Sprayer type, manufacturer, model | Nanospray Flex Ion Source (Thermo Scientific) |
| Other parameters if discriminant for the experiment | none |
| **2. Post source component — 3.1 Analyser** |  |
| Ion optics, ‘simple’ quadrupole, hexapole, Paul trap, linear trap, magnetic sector, FT-ICR, Orbitrap: name of the analyser(s) | MS1 survey scans in Orbitrap and MS2 analysed in LTQ ion trap |
| Time-of-Flight drift tube: Reflectron status | no TOF used |
| **3. Post-source component — 3.2 Activation / dissociation** |  |
| Instrument component where the activation /dissociation occurs | collision-induced dissociation (CID) in ion trap |
| Gas type (when used) | Helium |
| Activation / dissociation type | CID |
| **4. Spectrum and peak list generation and annotation — 4.1 Data acquisition** |  |
| Software name and version | LTQ Tune Plus Version 2.6.0 1065 SP3 (Thermo Scientific) HPLC Software : Chromeleon (Dionex Corporation), Chromeleon Client Version 6.80 |
| Acquisition parameters | The MS1 scan (m/z 400 to 1400) was acquired in the Orbitrap with 10^6^ ions (maximum filling time of 500 ms) and a resolution set to 60,000. Fragmentation was performed in the LTQ iontrap by collision-induced dissociation at 35 eV collision energy selecting the 10 most intense precursor ions (top10) with an isolation window of 3 m/z units, fragments were measured with a resolution of 7,500 in the Orbitrap. Target ions previously selected for fragmentation were dynamically excluded for 180 s with a relative mass window of 5 ppm. |
| **4. Spectrum and peak list generation and annotation — 4.2 Data analysis** |  |
| Software name and version | Manual data analysis: Qual Browser as part of Xcalibur 2.2 SP1.48 (Thermo Scientific) Automatic data analysis: PEAKS Studio 6.0 |
| **4. Spectrum and peak list generation and annotation — 4.3 Resulting data** |  |
| Location of source (‘raw’) and processed files | - |
| The chromatogram(s) for SRM data and other relevant cases | - |
| m/z and intensity values | see spectra |
| MS level | MS1 and MS2 |
| Ion mode | positive |
| For MS level 2 and higher, precursor m/z and charge if known, with the full mass spectrum / peaklist containing that precursor peak, where available | see corresponding spectra |

Table S 4 List of peptides found by nanoUHPLC-ESI-MS/MS protein identification experiments of purified recombinant AUS1. Score: 490 (-10lgP), sequence coverage: 78 %.

| **start-end** | **sequence** | **-10lgP/score** | **mass** | **ppm** | **m/z** |
| --- | --- | --- | --- | --- | --- |
| **2-16** | M.ALAPITAPDITSIC(+57.02)K.D | 62.28 | 1569.8436 | 0.2 | 785.9293 |
| **4-16** | L.APITAPDITSIC(+57.02)K.D | 21.99 | 1385.7224 | 2.0 | 693.8699 |
| **9-16** | A.PDITSIC(+57.02)K.D | 43.82 | 932.4637 | -0.8 | 467.2387 |
| **17-30** | K.DASSGIGNQEGAIR.T | 85.77 | 1373.6534 | -1.1 | 687.8333 |
| **18-30** | D.ASSGIGNQEGAIR.T | 56.07 | 1258.6266 | -0.5 | 630.3203 |
| **19-30** | A.SSGIGNQEGAIR.T | 55.71 | 1187.5895 | -1.1 | 594.8013 |
| **20-30** | S.SGIGNQEGAIR.T | 52.26 | 1100.5574 | -1.2 | 551.2853 |
| **22-30** | G.IGNQEGAIR.T | 35.40 | 956.5039 | 0.8 | 479.2596 |
| **33-41** | R.KC(+57.02)C(+57.02)PPSLGK.K | 53.36 | 1045.5049 | 0.3 | 523.7599 |
| **34-41** | K.C(+57.02)C(+57.02)PPSLGK.K | 28.16 | 917.4099 | 1.6 | 459.7130 |
| **43-53** | K.IKDFQFPNDKK.V | 54.58 | 1378.7245 | -1.2 | 690.3687 |
| **43-52** | K.IKDFQFPNDK.K | 54.47 | 1250.6295 | -1.3 | 626.3212 |
| **43-50** | K.IKDFQFPN.D | 30.78 | 1007.5076 | -1.1 | 504.7605 |
| **45-53** | K.DFQFPNDKK.V | 50.28 | 1137.5454 | -1.2 | 569.7793 |
| **45-52** | K.DFQFPNDK.K | 48.63 | 1009.4504 | -0.9 | 1010.4568 |
| **80-87** | R.ALPDDDPR.S | 42.70 | 897.4192 | -1.6 | 449.7161 |
| **120-129** | H.NSWLFFPFHR.W | 43.09 | 1349.6669 | -1.2 | 450.8957 |
| **130-137** | R.WYLYFYER.I | 60.29 | 1238.5760 | -2.2 | 620.2939 |
| **130-134** | R.WYLYF.Y | 20.88 | 790.3690 | -2.5 | 791.3743 |
| **130-135** | R.WYLYFY.E | 23.71 | 953.4323 | -0.5 | 477.7232 |
| **132-137** | Y.LYFYER.I | 37.86 | 889.4333 | -1.6 | 445.7232 |
| **138-147** | R.ILGSLIDEPN.F | 41.71 | 1069.5656 | -1.0 | 535.7895 |
| **137-148** | R.ILGSLIDEPNF.A | 52.28 | 1216.6339 | -0.6 | 609.3239 |
| **138-154** | R.ILGSLIDEPNFALPYWK.W | 96.22 | 1975.0454 | -2.8 | 659.3539 |
| **138-160** | R.ILGSLIDEPNFALPYWKWDEPKG.M | 66.82 | 2687.3635 | 0.3 | 896.7953 |
| **138-152** | R.ILGSLIDEPNFALPY.W | 66.95 | 1660.8712 | -1.1 | 554.6304 |
| **142-154** | S.LIDEPNFALPYWK.W | 69.69 | 1604.8239 | -1.7 | 803.4178 |
| **148-154** | N.FALPYWK.W | 37.10 | 923.4905 | -1.0 | 462.7520 |
| **155-159** | K.WDEPK.G | 24.33 | 673.3071 | 0.0 | 674.3144 |
| **160-165** | K.GMPISN.I | 15.66 | 617.2843 | 0.1 | 618.2916 |
| **160-180** | K.GM(+15.99)PISNIFLGDASNPLYDQYR.D | 117.87 | 2386.1262 | -1.5 | 796.3815 |
| **160-167** | K.GMPISNIF.L | 36.49 | 877.4368 | -0.9 | 439.7253 |
| **160-180** | K.GMPISNIFLGDASNPLYDQYR.D | 114.22 | 2370.1313 | -0.2 | 791.0508 |
| **160-173** | K.GMPISNIFLGDASN.P | 57.95 | 1434.6813 | -0.5 | 718.3475 |
| **162-180** | M.PISNIFLGDASNPLYDQYR.D | 89.76 | 2182.0693 | -0.3 | 728.3635 |
| **166-180** | N.IFLGDASNPLYDQYR.D | 91.05 | 1770.8577 | -1.4 | 886.4348 |
| **168-180** | F.LGDASNPLYDQYR.D | 79.80 | 1510.7052 | -1.1 | 756.3590 |
| **174-180** | N.PLYDQYR.D | 43.74 | 953.4606 | -1.7 | 477.7368 |
| **181-188** | R.DANHIEDR.I | 37.90 | 968.4312 | 1.9 | 485.2238 |
| **189-199** | R.IVDLDYDGKDK.D | 77.91 | 1279.6295 | -1.3 | 640.8212 |
| **189-197** | R.IVDLDYDGK.D | 68.97 | 1036.5077 | -1.9 | 519.2601 |
| **189-210** | R.IVDLDYDGKDKDIPDQQQVAC(+57.02)N.L | 68.02 | 2548.1750 | -1.2 | 850.3979 |
| **189-208** | R.IVDLDYDGKDKDIPDQQQVA.C | 51.48 | 2274.1016 | -1.5 | 759.0400 |
| **189-209** | R.IVDLDYDGKDKDIPDQQQVAC(+57.02).N | 75.97 | 2434.1321 | -1.7 | 812.3832 |
| **189-216** | R.IVDLDYDGKDKDIPDQQQVAC(+57.02)NLSTVYR.D | 96.89 | 3267.5718 | 1.9 | 1090.2000 |
| **190-199** | I.VDLDYDGKDK.D | 40.37 | 1166.5455 | -1.5 | 584.2792 |
| **191-197** | V.DLDYDGK.D | 28.34 | 824.3552 | -1.6 | 825.3611 |
| **198-210** | K.DKDIPDQQQVAC(+57.02)N.L | 69.98 | 1529.6780 | -0.6 | 765.8458 |
| **198-208** | K.DKDIPDQQQVA.C | 43.89 | 1255.6044 | -1.4 | 628.8086 |
| **198-209** | K.DKDIPDQQQVAC(+57.02).N | 60.87 | 1415.6350 | -1.5 | 708.8237 |
| **198-211** | K.DKDIPDQQQVAC(+57.02)NL.S | 69.77 | 1642.7621 | -2.5 | 822.3862 |
| **198-216** | K.DKDIPDQQQVAC(+57.02)NLSTVYR.D | 121.76 | 2249.0747 | -1.1 | 750.6981 |
| **200-216** | K.DIPDQQQVAC(+57.02)NLSTVYR.D | 94.13 | 2005.9528 | -1.6 | 1003.9821 |
| **210-216** | C.NLSTVYR.D | 29.74 | 851.4501 | -0.4 | 426.7321 |
| **211-216** | N.LSTVYR.D | 23.79 | 737.4072 | -0.9 | 738.4138 |
| **221-232** | R.NGVDPTSFFGGK.Y | 80.89 | 1224.5775 | -1.2 | 613.2953 |
| **222-232** | N.GVDPTSFFGGK.Y | 47.73 | 1110.5345 | -1.4 | 556.2737 |
| **233-260** | K.YVAGDSPVANGDPSVGSVEAGSHTAVHR.W | 92.33 | 2735.2898 | -1.3 | 912.7693 |
| **233-255** | K.YVAGDSPVANGDPSVGSVEAGSH.T | 96.44 | 2170.9766 | -1.1 | 724.6653 |
| **233-253** | K.YVAGDSPVANGDPSVGSVEAG.S | 65.35 | 1946.8857 | -1.5 | 974.4487 |
| **238-260** | D.SPVANGDPSVGSVEAGSHTAVHR.W | 71.37 | 2230.0725 | -1.1 | 558.5248 |
| **243-260** | N.GDPSVGSVEAGSHTAVHR.W | 61.37 | 1761.8394 | -1.4 | 441.4665 |
| **245-260** | D.PSVGSVEAGSHTAVHR.W | 87.41 | 1589.7910 | -0.1 | 530.9376 |
| **261-294** | R.WVGDPTQPNNEDM(+15.99)GNFYSAGYDPVFYIHHANVDR.M | 69.70 | 3940.7175 | -2.0 | 986.1847 |
| **261-294** | R.WVGDPTQPNNEDMGNFYSAGYDPVFYIHHANVDR.M | 75.74 | 3924.7227 | -0.9 | 982.1870 |
| **261-277** | R.WVGDPTQPNNEDMGNFY.S | 55.50 | 1982.8104 | -1.2 | 992.4113 |
| **261-288** | R.WVGDPTQPNNEDM(+15.99)GNFYSAGYDPVFYIH.H | 69.36 | 3248.3821 | 1.0 | 1083.8025 |
| **261-288** | R.WVGDPTQPNNEDMGNFYSAGYDPVFYIH.H | 80.05 | 3232.3872 | 0.9 | 1078.4707 |
| **261-286** | R.WVGDPTQPNNEDM(+15.99)GNFYSAGYDPVFY.I | 62.92 | 2998.2393 | -1.0 | 1000.4193 |
| **261-286** | R.WVGDPTQPNNEDMGNFYSAGYDPVFY.I | 66.13 | 2982.2441 | 0.2 | 995.0888 |
| **274-294** | M.GNFYSAGYDPVFYIHHANVDR.M | 65.25 | 2441.1189 | -1.2 | 611.2863 |
| **276-294** | N.FYSAGYDPVFYIHHANVDR.M | 55.15 | 2270.0544 | -3.1 | 568.5191 |
| **277-294** | F.YSAGYDPVFYIHHANVDR.M | 67.28 | 2122.9861 | -0.5 | 531.7535 |
| **278-294** | Y.SAGYDPVFYIHHANVDR.M | 67.82 | 1959.9227 | -1.3 | 654.3140 |
| **283-294** | D.PVFYIHHANVDR.M | 65.52 | 1466.7418 | -1.5 | 489.9205 |
| **304-312** | R.LPGHVDITD.P | 24.70 | 965.4818 | -2.8 | 483.7468 |
| **304-317** | R.LPGHVDITDPDWLN.A | 54.59 | 1590.7678 | -1.0 | 796.3904 |
| **304-319** | R.LPGHVDITDPDWLNAS.Y | 61.27 | 1748.8369 | -1.5 | 875.4244 |
| **304-316** | R.LPGHVDITDPDWL.N | 46.61 | 1476.7249 | -1.2 | 739.3688 |
| **304-331** | R.LPGHVDITDPDWLNASYVFYDENKDLVR.V | 120.99 | 3290.5884 | -2.8 | 1097.8671 |
| **304-327** | R.LPGHVDITDPDWLNASYVFYDENK.D | 94.26 | 2807.3079 | 0.1 | 936.7767 |
| **304-322** | R.LPGHVDITDPDWLNASYVF.Y | 41.42 | 2158.0371 | -1.8 | 1080.0239 |
| **304-323** | R.LPGHVDITDPDWLNASYVFY.D | 35.41 | 2321.1003 | -2.8 | 1161.5542 |
| **306-331** | P.GHVDITDPDWLNASYVFYDENKDLVR.V | 65.69 | 3080.4514 | -1.6 | 771.1189 |
| **306-327** | P.GHVDITDPDWLNASYVFYDENK.D | 86.94 | 2597.1709 | -1.8 | 866.7294 |
| **308-331** | H.VDITDPDWLNASYVFYDENKDLVR.V | 59.06 | 2886.3711 | -0.4 | 963.1306 |
| **313-331** | D.PDWLNASYVFYDENKDLVR.V | 81.35 | 2343.1172 | -2.0 | 782.0448 |
| **313-327** | D.PDWLNASYVFYDENK.D | 78.28 | 1859.8365 | -1.2 | 930.9244 |
| **317-327** | L.NASYVFYDENK.D | 59.23 | 1348.5935 | -1.4 | 675.3031 |
| **317-331** | L.NASYVFYDENKDLVR.V | 27.05 | 1831.8740 | -1.9 | 611.6308 |
| **318-327** | N.ASYVFYDENK.D | 56.59 | 1234.5505 | -1.9 | 618.2814 |
| **318-331** | N.ASYVFYDENKDLVR.V | 66.08 | 1717.8311 | -0.9 | 573.6171 |
| **320-327** | S.YVFYDENK.D | 17.20 | 1076.4814 | -0.9 | 539.2475 |
| **332-342** | R.VYNKDC(+57.02)VNLDK.L | 68.17 | 1366.6550 | -2.9 | 684.3328 |
| **336-342** | K.DC(+57.02)VNLDK.L | 40.87 | 862.3854 | 0.6 | 863.3932 |
| **345-352** | K.YNFIENSK.E | 56.02 | 1013.4818 | -2.0 | 1014.4874 |
| **345-350** | K.YNFIEN.S | 16.05 | 798.3548 | -1.2 | 799.3611 |
| **345-359** | K.YNFIENSKEVFPWRN.S | 34.12 | 1941.9373 | -0.9 | 648.3191 |
| **353-358** | K.EVFPWR.N | 32.38 | 832.4232 | -2.6 | 833.4282 |
| **367-378** | R.KSAQVATTGDVK.T | 37.68 | 1203.6459 | 2.7 | 402.2236 |
| **368-378** | K.SAQVATTGDVK.T | 72.91 | 1075.5509 | -0.1 | 538.7827 |
| **379-388** | K.TVEQTKFPVR.L | 63.24 | 1203.6611 | -0.6 | 602.8375 |
| **380-388** | T.VEQTKFPVR.L | 28.67 | 1102.6134 | -0.9 | 552.3135 |
| **389-394** | R.LNQIFK.V | 30.10 | 761.4435 | -2.0 | 762.4493 |
| **405-419** | R.TEEEKDQANEVLLIK.K | 94.92 | 1757.9047 | -2.0 | 879.9578 |
| **405-420** | R.TEEEKDQANEVLLIKK.I | 61.16 | 1885.9996 | -1.6 | 472.5064 |
| **405-417** | R.TEEEKDQANEVLL.I | 48.98 | 1516.7256 | -1.4 | 759.3690 |
| **410-419** | K.DQANEVLLIK.K | 58.36 | 1141.6343 | -0.9 | 571.8239 |
| **431-440** | K.FDVFVNDKLK.D | 59.39 | 1223.6550 | -1.3 | 408.8917 |
| **431-438** | K.FDVFVNDK.L | 52.01 | 982.4760 | -1.3 | 492.2446 |
| **439-464** | K.LKDGVFTTPC(+57.02)DPEYAGGFAQIPHNDK.K | 81.81 | 2876.3438 | -2.2 | 720.0916 |
| **439.461** | K.LKDGVFTTPC(+57.02)DPEYAGGFAQIPH.N | 90.10 | 2519.1790 | -1.4 | 840.7324 |
| **441-464** | K.DGVFTTPC(+57.02)DPEYAGGFAQIPHNDK.K | 55.62 | 2635.1648 | -1.8 | 879.3939 |
| **441-461** | K.DGVFTTPC(+57.02)DPEYAGGFAQIPH.N | 86.49 | 2278.0000 | -0.6 | 760.3401 |
| **441-456** | K.DGVFTTPC(+57.02)DPEYAGGF.A | 49.82 | 1731.7086 | -1.0 | 866.8607 |
| **466-474** | K.SM(+15.99)VMTSTAR.F | 55.95 | 998.4525 | 0.4 | 500.2337 |
| **466-474** | K.SM(+15.99)VM(+15.99)TSTAR.F | 33.57 | 1014.4474 | 2.8 | 508.2324 |
| **466-474** | K.SMVMTSTAR.F | 59.29 | 982.4576 | -3.5 | 492.2343 |
| **475-485** | R.FGLNELLEDTN.T | 38.97 | 1263.5983 | 0.2 | 632.8065 |
| **475-488** | R.FGLNELLEDTNTEG.E | 55.90 | 1550.7100 | 0.8 | 776.3629 |
| **475-499** | R.FGLNELLEDTNTEGEEYATVTLVPR.T | 88.05 | 2809.3657 | -0.2 | 703.3486 |
| **479-499** | N.ELLEDTNTEGEEYATVTLVPR.T | 65.01 | 2378.1489 | 0.3 | 793.7238 |
| **486-499** | N.TEGEEYATVTLVPR.T | 66.94 | 1563.7781 | -3.0 | 782.8940 |
| **500-511** | R.TGC(+57.02)EDLTVGEIK.I | 74.31 | 1320.6232 | -2.5 | 661.3172 |
| **500-511** | R.TGCEDLTVGEIK.I | 71.04 | 1263.6017 | -3.6 | 632.8058 |
| **503-511** | C.EDLTVGEIK.I | 28.12 | 1002.5233 | -1.2 | 502.2683 |

**Table S 5 List of most prominent proteins found by nanoUHPLC-ESI-MS/MS protein identification experiments of purified recombinant AUS1.**

| **species** | **unique peptides detected** | **sequence coverage** | **-10lgP/score** |
| --- | --- | --- | --- |
| AUS1\|KC972611 | 121 | 78 % | 489.68 |
| P36649\|CUEO_ECOLI Blue copper oxidase CueO OS=Escherichia coli (strain K12) | 10 | 34 % | 248.43 |
| B7LV13\|KAD_ESCF3 Adenylate kinase OS=Escherichia fergusonii | 11 | 43 % | 203.86 |
| P77202\|DSBG_ECOLI Thiol:disulfide interchange protein DsbG OS=Escherichia coli (strain K12) | 4 | 24 % | 186.74 |
| A4TPA4\|KAD_YERPP Adenylate kinase OS=Yersinia pestis (strain Pestoides F) | 6 | 26 % | 158.89 |
| P0C8Z2\|OMPA_ESCFE Outer membrane protein A (Fragment) OS=Escherichia fergusonii | 3 | 14 % | 139.44 |
| P0AFX0\|HPF_ECOLI Ribosome hibernation promoting factor OS=Escherichia coli (strain K12) | 4 | 35 % | 139.18 |

**Table S 6 Predicted results of transit peptide analysis by TargetP 1.1 (prediction of localization for cTP a chloroplast transit peptide, mTP a mitochondrial targeting peptide, SP a signal peptide) based on http://www.cbs.dtu.dk/services/TargetP/ (Emanuelsson et al. 2000).**

| **UniProt name** | **full-length amino acid sequence** | **cTP** | **mTP** | **SP** | **other** | **location** | **RC** |
| --- | --- | --- | --- | --- | --- | --- | --- |
| cgAUS1 | 602 | 0.896 | 0.084 | 0.011 | 0.186 | chloroplast | 2 |
| cgAUS2 | 615 | 0.951 | 0.074 | 0.048 | 0.047 | chloroplast | 1 |
| Q9FRX6_ANTMA | 562 | 0.048 | 0.096 | 0.022 | 0.946 | - | 1 |
| I7HUF2_TAROF | 600 | 0.814 | 0.166 | 0.023 | 0.094 | chloroplast | 2 |
| Q9ZP19_IPOBA | 496 | no transit peptide sequence information available in UniProt | | | | | |
| Q9MB14_IPOBA | 588 | 0.924 | 0.103 | 0.010 | 0.080 | chloroplast |  |
| P43311_PPO_VITVI | 607 | 0.928 | 0.034 | 0.014 | 0.085 | chloroplast | 1 |

**Table S 7 Predicted results of transit peptide analysis by ChloroP 1.1 (cTP tells whether or not this is predicted as a cTP-containing sequence; "Y" means that the sequence is predicted to contain a cTP; "-" means that is predicted not to contain a cTP.) based on http://www.cbs.dtu.dk/services/ChloroP/ (Emanuelsson et al. 1999).**

| **UniProt name** | **full-length amino acid sequence** | **score** | **cTP** | **CS-score** | **cTP-length** |
| --- | --- | --- | --- | --- | --- |
| AUS1 | 602 | 0.571 | Y | 1.542 | 35 |
| AUS2 | 615 | 0.561 | Y | 1.777 | 41 |
| Q9FRX6_ANTMA | 562 | 0.449 | - | -0.450 | 54 |
| I7HUF2_TAROF | 600 | 0.528 | Y | 7.795 | 34 |
| Q9ZP19_IPOBA | no transit peptide sequence information available in UniProt | | | | |
| Q9MB14_IPOBA | 588 | 0.564 | Y | 6.023 | 50 |
| P43311_PPO_VITVI | 607 | 0.581 | Y | 6.236 | 51 |
